# Identification of *O*-Glcnacylated Proteins in *Trypanosoma cruzi*

**DOI:** 10.3389/fendo.2019.00199

**Published:** 2019-03-29

**Authors:** Elia Torres-Gutiérrez, Yobana Pérez-Cervera, Luc Camoin, Edgar Zenteno, Moyira Osny Aquino-Gil, Tony Lefebvre, Margarita Cabrera-Bravo, Olivia Reynoso-Ducoing, Martha Irene Bucio-Torres, Paz María Salazar-Schettino

**Affiliations:** ^1^Facultad de Medicina, Universidad Nacional Autónoma de México, Ciudad de México, Mexico; ^2^Centro de Investigación Facultad de Medicina-UNAM and Facultad de Odontología, Universidad Autónoma Benito Juárez de Oaxaca, Oaxaca, Mexico; ^3^INSERM, Institut Paoli-Calmetes, CRCM, Marseille Protéomique, Aix-Marseille Univ, Marseille, France; ^4^Instituto Tecnológico de Oaxaca, Tecnológico Nacional de Mexico, Oaxaca, Mexico; ^5^CNRS, UMR 8576, UGSF, Unité de Glycobiologie Structurale et Fonctionnelle, Université de Lille, Lille, France

**Keywords:** *Trypanosoma cruzi*, O-GlcNAcylated proteins, post translational modification, epimastigote, protist, click chemistry, mass spectrometry

## Abstract

Originally an anthropozoonosis in the Americas, Chagas disease has spread from its previous borders through migration. It is caused by the protozoan *Trypanosoma cruzi*. Differences in disease severity have been attributed to a natural pleomorphism in *T. cruzi*. Several post-translational modifications (PTMs) have been studied in *T. cruzi*, but to date no work has focused on O-GlcNAcylation, a highly conserved monosaccharide-PTM of serine and threonine residues mainly found in nucleus, cytoplasm, and mitochondrion proteins. O-GlcNAcylation is thought to regulate protein function analogously to protein phosphorylation; indeed, crosstalk between both PTMs allows the cell to regulate its functions in response to nutrient levels and stress. Herein, we demonstrate O-GlcNAcylation in *T. cruzi* epimastigotes by three methods: by using specific antibodies against the modification in lysates and whole parasites, by click chemistry labeling, and by proteomics. In total, 1,271 putative O-GlcNAcylated proteins and six modification sequences were identified by mass spectrometry (data available via ProteomeXchange, ID PXD010285). Most of these proteins have structural and metabolic functions that are essential for parasite survival and evolution. Furthermore, O-GlcNAcylation pattern variations were observed by antibody detection under glucose deprivation and heat stress conditions, supporting their possible role in the adaptive response. Given the numerous biological processes in which O-GlcNAcylated proteins participate, its identification in *T. cruzi* proteins opens a new research field in the biology of Trypanosomatids, improve our understanding of infection processes and may allow us to identify new therapeutic targets.

## Introduction

The protozoan *Trypanosoma cruzi* is the causative agent of Chagas disease (CD). Also called American trypanosomiasis, CD is one of the biggest public health problems in Latin America. CD has spread to other continents due to increased population movements to and from Latin America. An estimated 8 million people are infected with the parasite worldwide (1). In 1909, the Brazilian physician Carlos Chagas described the disease in its acute and chronic phases. Most chronic-phase CD patients are symptom-free, but some may progress to cardiac, digestive, and/or neurological forms of the disease, which can be life-threatening when left untreated. The current treatment of CD is based on nifurtimox and benznidazole; developed in the 1960s and early 1970s. Both drugs have limitations, including a variable efficacy, long treatment courses, and toxicity. With only two drugs available for treatment, it is crucial to search for alternative targets for anti-CD therapies (2, 3). To this end, further information about basic regulatory functions in the parasite life cycle is much needed.

*Trypanosoma cruzi* is a protozoan parasite with a complex life cycle that requires one mammal and one arthropod host and involves three developmental stages. These changes allow the parasite to face environmental conditions such as variable temperatures and nutrient availability (4–6). The mechanisms that allow parasites to sense environmental changes and trigger a response are vital for their survival and establishment in a host (6, 7). The adaptive response requires modulating protein expression profiles, which are mainly regulated by post-transcriptional and post-translational modifications (PTMs) (8). Several PTMs have been reported in *T. cruzi*, including glycosylation of membrane proteins (9), acetylation of tubulins, and histone (10, 11), ubiquitination (12), SUMOylation (13), and phosphorylation of hundreds of proteins involved in several biological processes (14). Nevertheless, *O*-GlcNAcylation has not been reported yet in *T. cruzi* nor any Kinetoplastid protist.

*O*-GlcNAcylation is a dynamic PTM of proteins from the nucleus, cytoplasm, and mitochondria; it is involved in many different cell fundamental processes. Addition and removal of *O*-GlcNAc to/from proteins in animals is mediated by the enzymes *O*-GlcNAc transferase (OGT), and *O*-GlcNAcase (OGA) (15). UDP-GlcNAc, the donor substrate of OGT, is the final product of the hexosamine biosynthetic pathway (HBP). *O*-GlcNAcylation is thought to regulate protein functions in an analogous manner to protein phosphorylation. The crosstalk of both PTMs allows the regulation of cellular functions in response to nutrient levels and stress (16).

Protein *O*-GlcNAcylation has been reported in multicellular organisms and in some prokaryotic cells, but their presence in protists is a rather neglected field. Banerjee et al. reported homolog genes for the *O*-GlcNAc cycling enzymes in *Giardia lamblia* and *Cryptosporidium parvum* (17), and Perez Cervera et al. demonstrated the presence of *O*-GlcNAc-modified proteins in *Toxoplasma gondii* and *Plasmodium falciparum* by Western blot with the specific anti- *O*-GlcNAc RL2 and CTD 110.6 antibodies. Some *O*-GlcNAcylated proteins have been identified in these parasites, thirteen from *P. falciparum*, including actin, myosin, and the heat-shock protein HSP70. *O*-GlcNAcylated HSP70 was also identified by immunoprecipitation in *T. gondii*, and recently, proteomic analyses based on s-WGA enrichment and click chemistry revealed 357 *O*-GlcNAcylated proteins with several functions, including rhoptries, that are necessary for invasion (18–20).

This work is aimed to demonstrate the presence and to visualize *O*-GlcNAc-modified proteins in *T. cruzi* epimastigotes, and to evaluate the influence of some environmental conditions on *O*-GlcNAcylation patterns by Western blot, immunofluorescence, and enzymatic labeling. The enrichment of *O*-GlcNAc proteins was improved by a click chemistry-based strategy using an alkyne resin. Then, 1,271 putative *O*-GlcNAcylated proteins and six modification sites were identified by MS/MS.

## Materials and Methods

### Culture of *Trypanosoma cruzi* Epimastigotes

*Trypanosoma cruzi* epimastigotes were cultured at 28°C in RPMI 1,640 medium supplemented with 10% heat-inactivated fetal bovine serum (FBS). Cultures were maintained in the growth phase.

Heat stress: *T. cruzi* epimastigotes were cultured in 25-cm^2^ dishes, in 10 mL of RPMI 1,640 medium either at 28 or 37°C, at a concentration of 10^6^ cells/mL. After 4 days of incubation, cultured cells were harvested by centrifugation at 2,500 × *g* for 20 min at 4°C and washed three times with PBS.

Glucose availability: Culture dishes with 10 mL of RPMI 1,640 medium supplemented with various glucose concentrations (17, 11.5, 5.5, and 0 mM) were inoculated with 10^6^ parasites/mL and incubated for 5 days. Then, the cells were harvested by centrifugation at 2,500 × g for 20 min at 4°C and washed three times with PBS.

### Protein Extraction

Control and experimental parasite cultures were lysed in the following homogenization buffer: 10 mM Tris/HCl, 150 mM NaCl, 1 mM EDTA, 1% (v/v) Triton X-100, 0.5% (w/v) sodium deoxycholate, 0.1% (w/v) SDS, protease inhibitor, pH 7.4. After centrifugation at 20,000 × g for 10 min, supernatants were recovered and frozen until used.

### SDS-PAGE and Western Blotting

Proteins were run on 12% SDS-PAGE under reducing conditions. Gels were either stained with Coomassie blue or electroblotted onto a PVDF sheet. Blots were saturated with 5% (w/v) blotting-grade blocker (Bio-Rad) in TBS (Tris-buffered saline)-Tween [15 mM Tris, 140 mM NaCl, 0.5% (v/v) Tween] for 30 min. Primary antibodies were incubated overnight at 4°C. Mouse monoclonal anti-*O*-GlcNAc RL2 (ab2739) was used at a 1:1,000 dilution; mouse polyclonal anti-*O*-GlcNAc CTD 110.6 and anti-alpha tubulin DM1A (Sigma, St Louis Missouri, USA) antibodies were also used. The specificity of the RL2 antibodies was checked by co-incubation with 1 M free *O*-GlcNAc. Then, the membranes were washed three times for 10 min with TBS-Tween and incubated with anti-mouse IgG or IgM, HRP labeled secondary antibodies (Abcam, Cambridge, UK) at a 1:5,000 dilution. The membranes were washed three times for 10 min with TBS-Tween, and spots were detected by enhanced chemiluminescence with Hyperfilms (GE Healthcare, Chicago, USA). Three independent experiments were performed and images were captured using a Bio Rad Gel Doc imaging system and processed with Quantity One software. One representative blot is shown.

### Immunoprecipitation

Immunoprecipitation protocol was carried on using magnetic beads (Bio-Rad California, USA). Ten microgram of anti-tubulin DM1A antibody (Sigma) diluted on 200 L of PBS-Tween 0.1% were incubated on Protein C magnetic beads for 10 min at room temperature, then magnetized and supernatant were discarded. After three washes, lysed epimastigotes were added and incubated 1 h. Then three washes were realized and beads transferred to a new tube. Finally, 40 μl of 1x reduced Laemmli Sample Buffer were added and incubated for 10 min at 70°C. SDS PAGE was performed and Wb using anti-*O*-GlcNAc RL2 as primary antibody, HRP conjugated anti-mouse as secondary antibody and revealed using HRP color development reagent (Bio-Rad).

### Immunofluorescence Microscopy

For immunolabeling, purified *T. cruzi* epimastigotes were fixed in 4% (m/v) paraformaldehyde in PBS for 1 h at room temperature and washed with PBS. Parasite cells were permeabilized with 0.1% Tween 20 for 90 min. Non-specific sites were blocked with 1% BSA. Anti *O*-GlcNAc antibodies RL2 diluted 1:50 (in PBS) were added and incubated overnight. After three washes with PBS, the parasites were incubated with anti-mouse FITC antibodies (1:100 in PBS) and then fixed on glass slides with Fluoro Shield DAPI (Sigma). A Leica DM2000 microscope with a Leica DFC310 FX camera was used for visualization. The images were processed with the software Image J. One representative figure is shown of three independent experiments.

### Enzymatic Labeling of O-GlcNAcylated Proteins

Click-it *O*-GlcNAc Enzymatic Labeling System (Invitrogen C33368) is a method for modification *in vitro* of *O*-GlcNAcylated proteins. Proteins were enzymatically labeled by the permissive mutant B-1,4 galactosyltransferase (Gal T1 Y289L), which transfers azido-modified galactose (GalNAz) from UDP-GalNAz to *O*-GlcNAc residues in the target proteins. A protein extract with no enzymatic treatment was used as a negative control, and α-crystallin, a protein with a low *O*-GlcNAcylation level (2–10%) was used as a positive control. Click-it *O*-GlcNAc enzymatic labeling was performed following the manufacturer's protocols. Labeled proteins were detected by Western blot with the Click-it Biotin Protein Analysis Detection Kit (Invitrogen C33372).

### O-GlcNAcylated Proteins Labeling and Enrichment

Click-it *O*-GlcNAc enzymatic labeling was performed on *T. cruzi* protein extracts following the manufacturer's protocols, as described by Hahne et al. (21).

*O*-GlcNAcylated *T. cruzi* epimastigote proteins with azide tag were then enriched by covalent capture onto an alkyne resin through click chemistry, using the Click-it Protein Enrichment Kit (Invitrogen C10416). This technique uses the Cu(I)-catalyzed Huisgen cycloaddition to promote a cyclic addition reaction between an azide and a terminal alkyne, generating a 1,4-disubstituted 1,2,3-triazole as a covalent linkage. When the click reaction was complete, the beads with *O*-GlcNAcylated proteins were first reduced with 10 mM dithiothreitol (DTT) for 30 min at 55°C and then alkylated with 50 mM iodoacetamide (IAA) for 60 min at room temperature. The resin was subjected to an extensive washing procedure in column as follows: five washes with 1.5 mL of SDS wash buffer (100 mM Tris/HCl, pH 8; 1% SDS; 250 mM NaCl; 5 mM EDTA); five washes with 1.5 mL of urea buffer (8 M urea; 100 mM Tris/HCl, pH 8); 10 washes with 1.5 mL of 20% acetonitrile (ACN); and two washes with 1 mL of digestion buffer (100 mM TEAB, pH 8.2; 10% ACN).

### Protein Digestion

Resin-bound proteins were digested overnight in 200 μL of digestion buffer containing 1 μg of trypsin/Lys-C mix. After digestion, the supernatant solution was discarded, and the resin was washed with 500 μL of deionized water. Both solutions, one containing non-retained peptides and the other containing *O*-GlcNAc proteins, were pooled and stored before desalting. The resin was then washed twice with 1.5 mL of MS-grade water, followed by two more washes with 1.5 mL of dephosphorylation buffer (50 mM Tris/HCl, pH 7.6; 100 mM NaCl; 1 mM DTT; 10 mM MgCl_2_; 1 mM MnCl_2_). Non-retained peptides were desalted in a C18 reversed-phase column and dried in a centrifugal vacuum system before LC-MS/MS analysis.

### Beta-Elimination

To confirm *O*-GlcNAc sites an on-resin dephosphorylation step between the on-resin proteolytic digest and the on-resin β-elimination was added. So ideally all peptides bound to the alkyne resin should be *O*-GlcNAc modified. *O*-GlcNAcylated peptides linked to agarose beads were dephosphorylated at 37°C for 6 h in 400 μL of dephosphorylation buffer using 800 U of λ phosphatase and 20 U of calf intestine phosphatase. After dephosphorylation, the resin was washed twice with 1.5 mL of water and the slurry volume was adjusted to 300 μL with water before treatment with the GlycoProfile β-elimination Kit (Sigma Aldrich). The reaction mixture was incubated in an end-over-end shaker with extensive mixing at 4°C and quenched after 24 h with 1% trifluoroacetic acid (TFA). Agarose beads were discarded, and the solution containing β-eliminated peptides, corresponding to *O*-GlcNAcylated peptides, was desalted in C18 reversed-phase columns and dried in a centrifugal vacuum system before LC-MS/MS analysis.

### Mass Spectrometry

The samples were reconstituted with 0.1% TFA in 4% ACN and analyzed by liquid chromatography (LC)-tandem mass spectrometry (MS/MS) using an Orbitrap Fusion Lumos Tribrid Mass Spectrometer (Thermo Electron, Bremen, Germany) online with an Ultimate 3000RSLCnano chromatographic system (Thermo Fisher Scientific, Sunnyvale, CA). The peptides were separated using a Dionex Acclaim PepMap RSLC C18 column. First, the peptides were concentrated and purified with a Dionex pre-column (C18 PepMap100, 2 cm × 100 μm ID, 100 Å pore size, 5 μm particle size) in solvent A (0.1% formic acid, 2% acetonitrile). Then, the peptides were separated on a Dionex reverse-phase LC EASY-Spray C18 column (PepMap RSLC C18, 50 cm × 75 μm ID, 100 Å pore size, 2 μm particle size) at a 300 nL/min flow rate and 40°C. After column equilibration using 4% of solvent B (20% water-80% ACN-0.1% formic acid), the peptides were eluted from the analytical column by a two-step linear gradient (4–20% ACN/H_2_O-0.1% formic acid for 220 min and 20–45% ACN/H_2_O-0.1% formic acid for 20 min). For peptide ionization, spray voltage was set at 2.2 kV and the capillary temperature at 275°C. The mass spectrometer was used in data-dependent mode to switch consistently between MS and MS/MS. The time between master scans was set to 3 s. MS spectra were acquired in an *m/z* range of 375–1,500, with a FWHM resolution of 120 000 measured at 200 *m/z*. AGC target was set at 4.0e5 with a maximum injection time of 50 ms. The ion generated from polydimethylcyclosiloxane during the electrospray process the protonated (Si(CH_3_)_2_O)_6_) at *m*/*z* 445.120025 was used as lock mass for internal mass calibration. The most abundant precursor ions were selected, and a higher-energy collisional dissociation fragmentation was performed and analyzed in the Orbitrap analyzer with a resolution of 50,000. The number of precursor ions was automatically defined along run in 3 s windows, using the Inject Ions for All Available Parallelizable Time option with a maximum injection time of 105 ms and an AGC target of 1.0e5. Charge state screening was enabled to include precursors with two and seven charge states. Dynamic exclusion was enabled with a repeat count of one and a duration of 60 s.

### Protein Identification and Abundance Quantification

The acquired raw MS data were processed with the software Proteome Discoverer v.1.4.1.14 (Thermo Fisher Scientific). Data were searched via SEQUEST HT against the Uniprot *T. cruzi* reference proteome database (retrieved on February 14, 2018, 44 286 entries). The following parameters were used for searches: (i) trypsin; (ii) two missed cleavages were allowed; (ii) monoisotopic precursor tolerance of 10 ppm, followed by 0.6 Da for fragment ions from MS/MS; and (iii) cysteine carbamidomethylation (+57.0215) and methionine oxidation (+15.995) as variable modifications. False discovery rate (FDR) was processed using Percolator a semi-supervised machine learning to discriminate correct from incorrect peptide-spectrum matches (22), and was set to a *q*-value of 1 and 5% for, respectively, define high and low confident peptides. In addition, only peptide spectrum matches with a delta Cn Sequest HT parameters better than 0.15 were considered and proteins were identified with at least two peptides per protein. Additionally, a threshold was established based on the MS area, which meant that the proteins with the lowest intensities which conformed the summed intensity of 7 × 10^8^ were considered potential background (21). The abundance of the different identified proteins were determined by label-free quantitative proteomics using the TOP 3 method (23, 24). Peptides from β-elimination experiments were identified as described above, except that dehydration of Ser and Thr (−18.011 a.m.u.) and β-elimination of Cys (−33.988 a.m.u.) were added as variable modifications. Mass spectrometry proteomics data were deposited in the ProteomeXchange Consortium via the PRIDE (25) partner repository with the dataset identifier PXD010285.

## Results

### *Trypanosoma cruzi* Express O-GlcNAcylated Proteins

**Figure 1:**
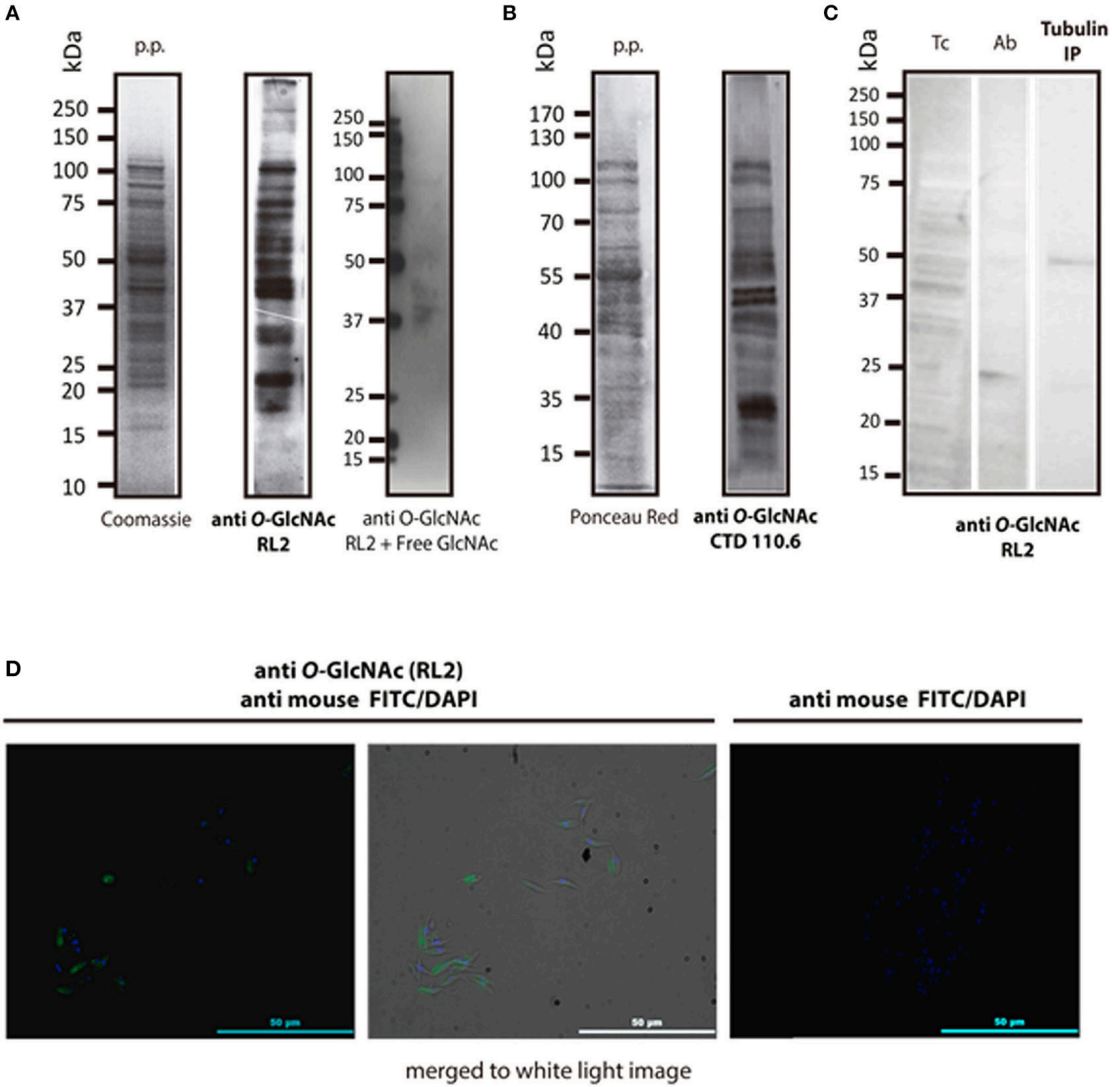
Immune detection of *O*-GlcNAcylated proteins from *T. cruzi* epimastigotes. **(A)** RL2 antibody. Protein profile (p.p.) of epimastigotes in SDS-PAGE Coomassie blue, 20 μg, and Western blot with the RL2 antibody and Free GlcNAc control. Visualized by chemiluminescence. **(B)** CTD 110.6 antibody. Protein profile (p.p.) of epimastigotes stained with Red Ponceau, 30 μg. B2, Western blot with CTD 110.6. Visualized by chemiluminescence. **(C)** *T. cruzi* Alpha tubulin immunoprecipitation (DM1A ab) in western blot with anti *O*-GlcNAc RL-2. Tc, epimastigote lisate; Ab, antibody DM1A Tubulin IP; Immunoprecipitated Tubulin. **(D)** Immunofluorescence microscopy. Anti-*O*-GlcNAc RL-2 was used as the primary antibody and FITC-labeled anti-mouse as the secondary antibody.

After the *O*-GlcNAc modification was first described in 1984, several approaches have been used to detect it. Antibody detection and residue enzymatic elongation by bovine GalT are commonly used. Both assays were performed herein to visualize *T. cruzi O*-GlcNAcome. A wide range of proteins, from 10 to 250 kDa, were recognized when epimastigote proteins were exposed to the broadly used anti *O*-GlcNAc antibodies RL2 (Figure 1A) and CTD110.6 (Figure 1B). *O*-GlcNAcylation was also detected in whole epimastigotes by immunofluorescence microscopy with the RL2 antibody (Figure 1D). To show evidence about the *O*-GlcNAcylation of an specific protein, an immunoprecipitation protocol were performed to isolate alpha tubulin, that were previously described in other protist parasites (19). The *O*-GlcNAcylation was revealed by western blot using RL2 as primary antibody.

**Figure 2:**
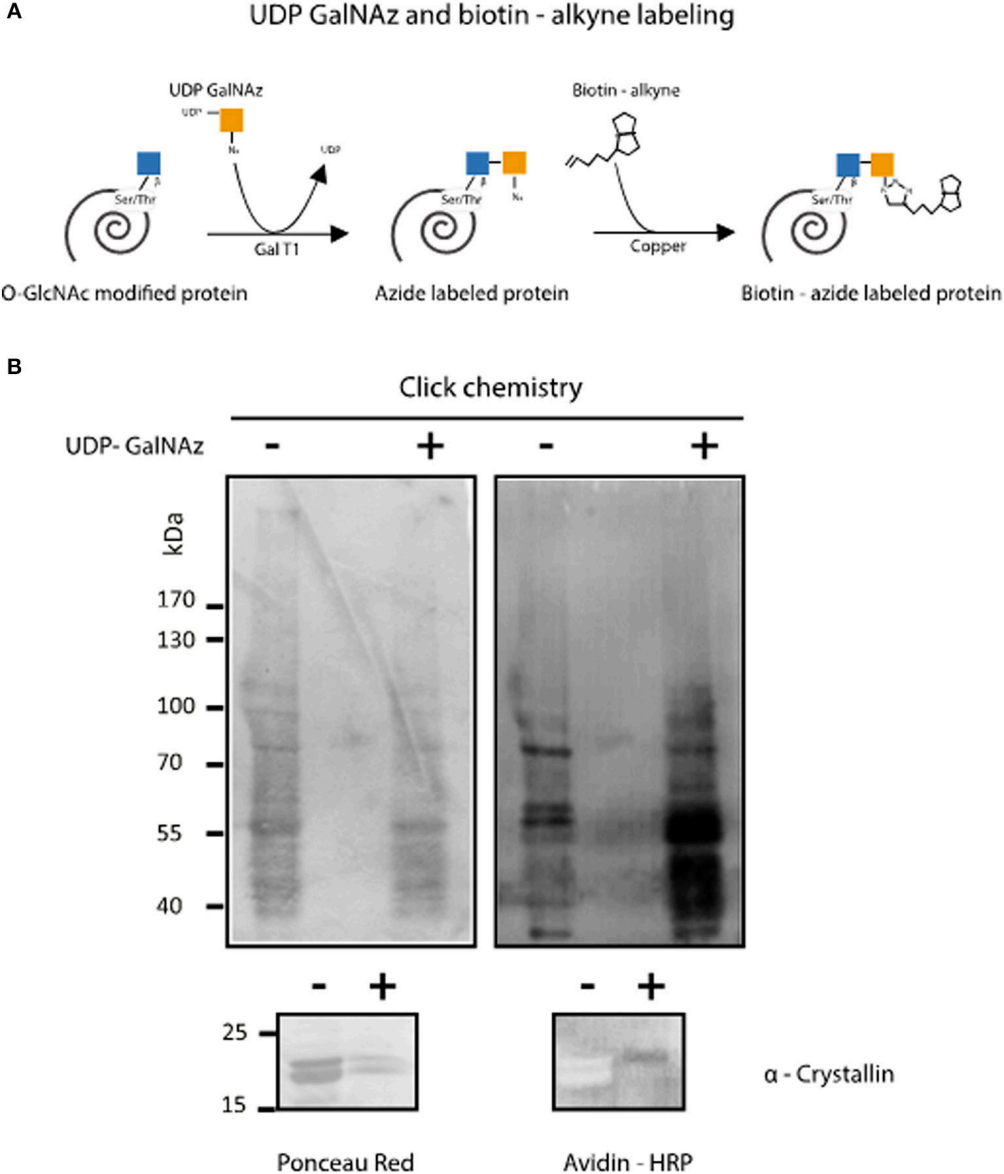
Enzymatic detection of *T. cruzi O*-GlcNAcilayted proteins by click chemistry. **(A)** *O*-GlcNAcylated protein labeling by GalNAz and biotin alkyne using the Click iT™ *O*-GlcNAc Enzymatic Labeling System and the Glycoprotein Detection Kit. **(B)** After labeling, *Trypanosoma cruzi O*-GlcNAcylated proteins were separated by SDS-PAGE, and Western blot was performed using HRP-labeled avidin. Protein load was assessed by Ponceau Red staining. α-Crystallin was used to control labeling efficiency.

To confirm the presence of *O*-GlcNAcylated proteins in *T. cruzi* epimastigotes, a galactose derivative (GalNAz) was bound to GlcNAc moieties by an engineered galactosyltransferase (GalT1 Y289L). This was followed by a selective and specific chemical addition of biotinalkyne to allow the detection of the tagged proteins or peptides by the avidin-peroxidase system (Figure 2A). This highly sensitive technique confirmed that the *T. cruzi* proteome is rich in *O*-GlcNAcylated proteins (Figure 2B).

These results demonstrate for the first time the presence of *O*-GlcNAc modifications in *T. cruzi*. No previous reports have been published about this PTM in any Kinetoplastid parasite.

### Environmental Conditions Influence Protein O-GlcNAcylation Pattern in *T. cruzi* Epimastigotes

**Figure 3:**
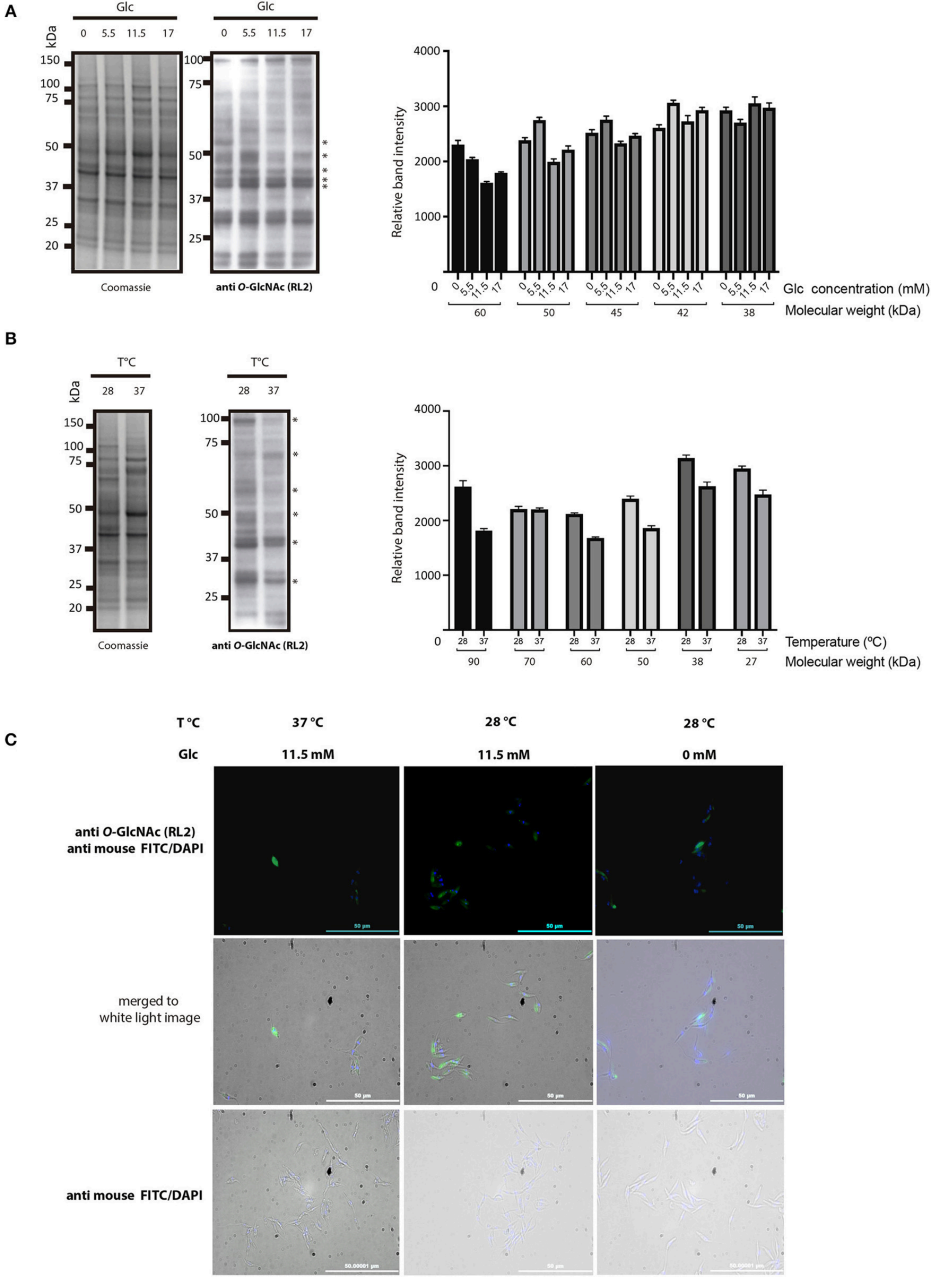
*Trypanosoma cruzi* epimastigotes *O*-GlcNAcylation under various glucose availability and heat stress conditions. **(A)** *O*-GlcNAcylation profile under glucose availability variations. Glucose availability in culture media was 0, 5.5, 11.5, and 17 mM. Densitometry of several bands (*). **(B)** *O*-GlcNAcylation profile under heat stress at 37°C (28°C was the normal culture temperature). Densitometry of several bands (*). **(C)** Immunofluorescence microscopy. 28°C and Glc 11.5 mM were normal culture conditions. Heat stress at 37°C and low Glc availability (0 mM). Anti *O*-GlcNAc RL-2 antibody was used as the primary antibody and FITC-labeled anti mouse as the secondary antibody.

Protein *O*-GlcNAcylation exhibits a great dynamism. To assess whether *O*-GlcNAcylation patterns vary under diverse environmental conditions, parasites were grown under different temperatures and glucose availability. While *O*-GlcNAcylation was detected by western blot and immunofluorescence in every condition (Figure 3C), *O*-GlcNAcylated protein patterns under low glucose availability (0–5.5 mM) showed a higher reaction in bands of 60, 50, and 45 kDa. The 42 kDa band show higher *O*-GlcNAcylation mainly on 5.5 mM glucose condition (Figure 3A). Contrasting, under heat stress (37°C) most components showed reduced *O*-GlcNAcylation, except for 70 kDa band, as shown in Figure 3B.

### Identification of O-GlcNAcylated Proteins in *T. cruzi*

**Figure 4:**
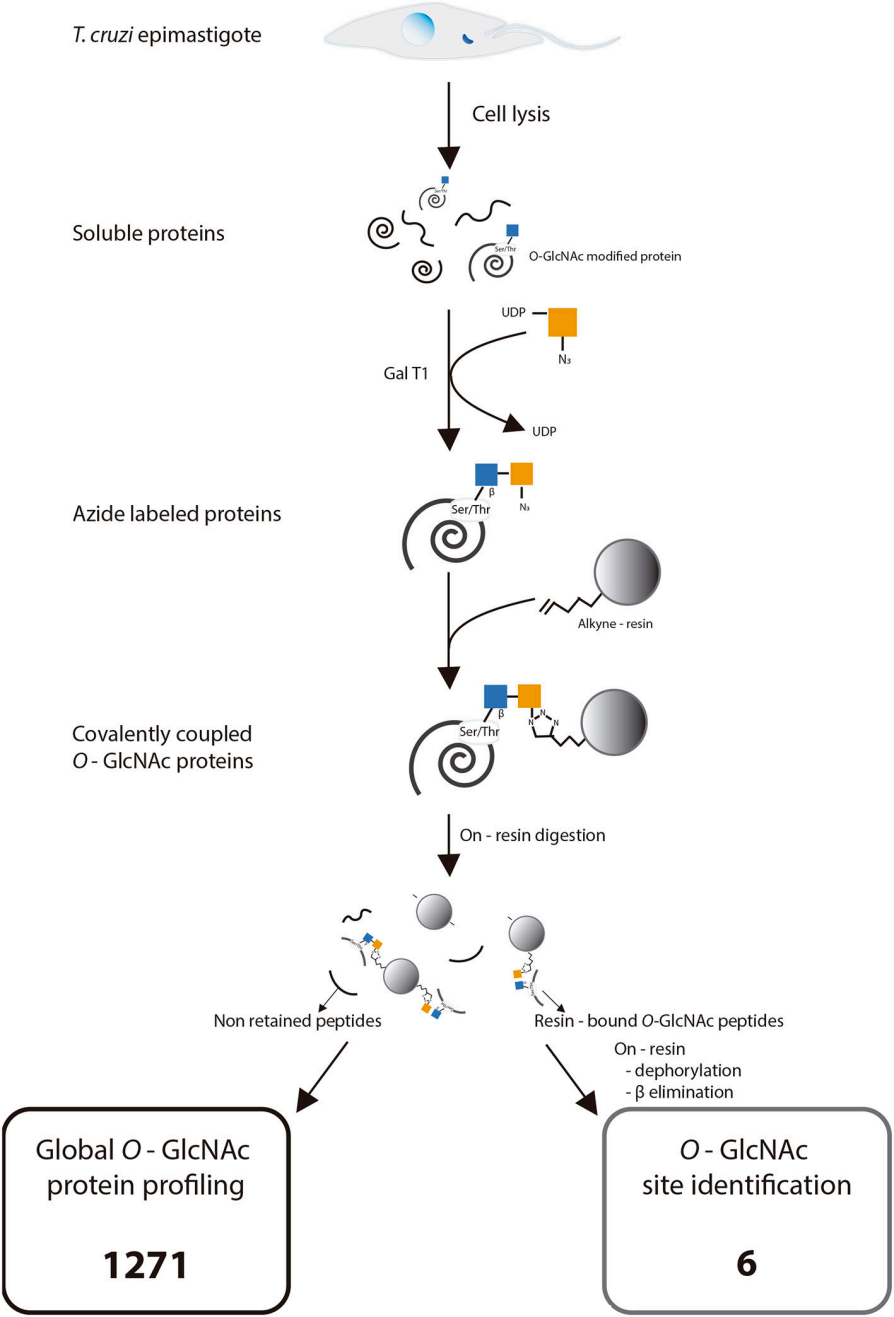
Experimental strategy for click chemistry-based labeling, enrichment, and identification of *Trypanosoma cruzi O*-GlcNAc modified proteins. Adapted with permission from Hahne et al. (21). Copyright 2013 American Chemical Society. ◦ **1271** putative proteins identified by Global *O*-GlcNAcylated protein profiling. ◦ **6** modification sites. Full data are available via ProteomeXchange with identifier PXD010285.

**Figure 5:**
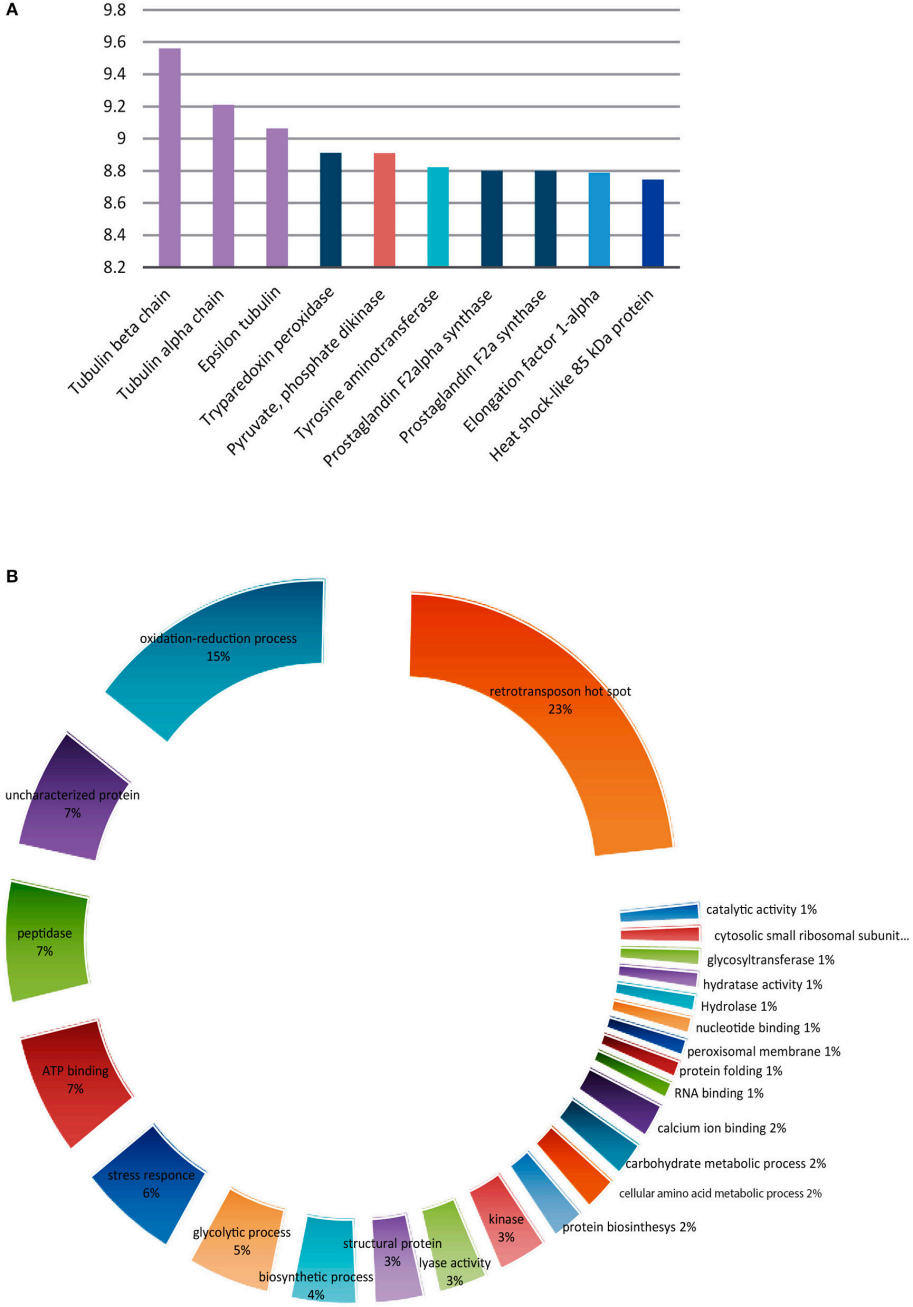
*Trypanosoma cruzi* putative O-GlcNAcylated proteins. **(A)** The 10 most abundant using the TOP three method. **(B)** Classification according to their function of the 100 most abundant.

Once the presence of *O*-GlcNAcylated proteins in *T. cruzi* was established, their identity was studied. Several methods for protein identification have been described, including some based on lectins or antibody enrichment; in the past 10 years, click chemistry-based methods have gained prominence. The highly sensitive labeling of proteins by azide-modified galactose and copper-mediated click chemistry was used herein, followed by purification of modified proteins on an alkyne-resin (17) (Figure 4). On-resin trypsin proteolysis followed by LC-MS/MS allowed the identification of 1,271 putative *O*-GlcNAc proteins at 5% false discovery rate (FDR) and eliminating the proteins with the lowest intensities which could be considered as potential background as mentioned in materials and methods. These proteins belong to a broad range of biological functions and participating in various cellular pathways. Of the 10 most abundant putative *O*-GlcNAcylated proteins, three are constitutive of the cytoskeleton, three participate in oxidation reduction processes and the others include kinases, and proteins that participate in biosynthesis and stress response. The classification by function of the 100 most abundant based on Top3 quantification, are shown in Figure 5, and full data are available via ProteomeXchange, ID PXD010285 and Supplementary Table 1. Subsequent elution of on-resin *O*-GlcNAcylated peptides by β-elimination led to the identification of 6 peptides corresponding to *O*-GlcNAc modification sites at 5% FDR (Supplementary Figure 1).

## Discussion

Protein *O*-GlcNAcylation is ubiquitous in pluricellular organisms and has also been described in simple organisms, including viruses (26) and bacteria (27). Nevertheless, there are only few publications about protein *O*-GlcNAcylation in protist organisms like *Giardia lamblia* and *Cryptosporidium parvum* (17), *Toxoplasma gondii* (18), and *Plasmodium falciparum* (19). To date, there is no report about protein *O*-GlcNAcylation in *T. cruzi* or any Kinetoplastid protist. In this work, we demonstrated the presence of *O*-GlcNAcylated proteins in *T. cruzi* epimastigotes and determined the identity of 1,271 putative *O*-GlcNAcylated proteins. Surprisingly, only 6 *O*-GlcNAc modifications sites were identified using dehydratation of Ser and Thr after beta-elimination from beads. MS-MS spectra of these peptides are given as Supplementary Figure 1. This low detection of modified peptides varies from our previous similar study (28). The explanation could be associated to the lower amount of proteins initially used and to the higher number of *O*-GlcNAc proteins. Each *O*-GlcNAc modified peptide is related to one of the following protein groups: clathrins, RNA helicases, DNA polymerases, trans-sialidases and two uncharacterized proteins as seen in Supplementary Table 2.

Several methods have been reported to detect *O*-GlcNAcylation. First, the sugar nucleotide UDP-GlcNAc is known to be a substrate for this PTM. UDP-GlcNAc is relatively abundant in *T. cruzi*, being produced by conventional HBP as previously reported by Turnock and Ferguson (29). Second, a search for the sequences of the regulatory enzymes OGT and OGA in *T. cruzi* genome databases (for instance, by Banerje et al.) has been unfruitful, maybe due to the great number of unknown proteins in the parasite genome and the fact that it is not completely cured. Nevertheless, some organisms have been reported as producing *O*-GlcNAcylated proteins, like the protist *P. falciparum*, whose OGT and OGA enzyme variants have not been characterized yet. Bacterial species like *Listeria monocytogenes* (30) and *Streptococcus pneumoniae* (31) code for different OGT variants, which are not homologous to the animal enzymes even when they have been proved to have critical roles in functions like motility and adhesion. All OGT-detecting methods rely on antibodies or activity measurement, even though antibody detection is only acceptable when the OGT coding gene has been fully identified.

This work is focused on detecting *O*-GlcNAcylated proteins by proteomic approaches (32). Various monoclonal antibodies that recognize *O*-GlcNAcylated proteins are available, including the RL2, CTD 110.6, 18B10.C7, 9D1.E4, and 1F5.D6 antibodies. The most widely used and more readily available antibodies to detect *O*-GlcNAcylation by Western blot are RL2 (33) and CTD 110.6 (34); these antibodies can detect a variety of modified proteins in different organisms, including protist cells (18, 19). Both antibodies were used herein to visualize *O*-GlcNAcylated protein in *T. cruzi* epimastigotes proteome; additionally, RL2 was used for fluorescence microscopy analysis. The parasite life cycle includes three development stages, and we focused our research on epimastigotes, being the only replicative extracellular stage, so we can prevent mammal cell contamination during protein extraction. As shown in Figure 1, the *T. cruzi* proteome is rich in *O*-GlcNAcylated proteins with a wide range of molecular weights, as previously observed for *T. gondii* and *P. falciparum*. RL2 is an IgG antibody that was raised specifically against *O*-GlcNAc moieties of nuclear pore complex (24). However, it can also detect some other *O*-GlcNAc modified proteins. CTD110.6 is an IgM antibody directed against the *O*-GlcNAcylated C-terminal domain of RNA polymerase II proteins (34). The latter has a broader reactivity than RL2, recognizing a variety of *O*-GlcNAc-modified proteins. These antibodies RL2 and CTD 110.6 did not recognize the same epitopes and therefore the observed patterns were not equal, as it has been previously reported (35). Western blot using RL2, still revealed that immunoprecipitated alpha tubulin was also *O*-GlcNAcylated, which have been reported for *P. falciparum* and *T. gondii* (19, 20). Immunofluorescence analysis with RL2 allowed us to visualize the *O*-GlcNac modification directly in the whole parasite. This first evidence of the presence of *O*-GlcNAcylated proteins in this parasite was further confirmed by a different approach. The *O*-GlcNAc modification (36) was first described by elongating *O*-GlcNAcylated residues with bovine GalT using UDP-[^3^H]-galactose; in the last 10 years, this method has evolved to include techniques from click chemistry, which use a engineered modified GalT (GalT1 Y289L) that transfers GalNAz to *O*-GlcNAcylated residues, followed by a selective addition of biotin alkyne. This approach demonstrated a rich pattern of protein bands in *T. cruzi* lysates (Figure 2), confirming the presence of this PTM in *T. cruzi*.

Since *O*-GlcNAcylation is characterized by a dynamic response to medium stimuli, we exposed parasites to different amounts of glucose, a nutrient required for UDP-GlcNAc production, also considering that nutrient deprivation triggers metacyclogenesis in the hindgut of the insect host. Heat stress is another environmental condition that *T. cruzi* faces during mammal infection. *O*-GlcNAcylation patterns as observed by immunofluorescence showed no significant variations (Figure 3C); in contrast, the level of reaction observed in 60–42 kDa components by Western blot was higher in protein extracts from the low-glucose group (0–5.5 mM) (Figure 3A) and in 70-kDa band in the group under heat stress (Figure 3B), which could correspond to one of the previously reported parasite heat-shock protein (HSP) (7) and suggest a possible role of *O*-GlcNAcylation in the cellular homeostatic response in *T. cruzi*.

Once the presence of *O*-GlcNAcylated proteins in *T. cruzi* was established, we focused on identifying them. Thousands of *O*-GlcNAcylated proteins have been reported in mammal cells; however, only few *O*-GlcNAcylated proteins have been identified in protist organisms; HSP70 and tubulin were confirmed in *T. gondii* by immunoprecipitation (18). The same proteins were identified in *P. falciparum* by a combination of click chemistry enrichment and specific HSP70 and tubulin immune detection. In the same work, WGA- and click chemistry-enriched extracts were resolved by SDS-PAGE; the bands were excised and analyzed by mass spectrometry, identifying 13 proteins that participate in cell functions like glycolysis and cytoskeleton organization, or as chaperones (19). A similar approach allowed the detection of 357 *O*-GlcNAcylated proteins in *T. gondii*, including rhoptries, which play a role in host infection (20). Considering the work by Kupfershmid and other authors, reporting that click chemistry-based strategies improved protein identification (21, 37, 38), we combined click chemistry-based *O*-GlcNAcylated protein labeling, click chemistry enrichment on alkyn resin, and on-resin trypsin proteolysis with MS/MS peptide sequencing. However, it must be noted that there is a potential interference in the enrichment procedure with non-reducing GlcNAc residues on *O*-linked and *N*-linked glycoproteins as have been reported for ovalbumin (39); when in a similar approach only 1% of the proteins were unspecifically bound, as the summed intensity of the labeled sample were 60-fold higher than the negative control (21), in the present work an additional threshold was set based on the MS area as described in the material and methods section. This combination of methods allowed us to identify 1,271 proteins displaying a wide variety of biological functions, including nucleic acid synthesis, transcription, protein synthesis, structural constitution of cytoskeleton, mitochondrial function, stress response, ATP cycling, peptidase activity and noteworthy retro-transposon hot spot (RHS) that are kinetoplastids exclusive proteins whose functions remain unveiled (data are available via ProteomeXchange, ID PXD010285 and Supplementary Table 1). It is well-known that the most abundant surface glycoproteins of *T. cruzi* are the mucin-like proteins, which can be modified with multiple glycan chains attached to the peptide by α-GlcNAc-*O*-Thr linkages (40, 41). Also when it is unusual for surface glycoproteins, in some strains, an amount of non-substituted *O*-linked GlcNAc has been reported (42). Many cell surface, lysosomal, and secreted proteins like trans-sialidases, mucins and some proteases, are post-translationally modified by the addition of a β-GlcNAc to the asparagine (Asn) residues, usually followed by complex branched high mannose glycans but it could also be found as a small chitobiose glycan (GlcNAc_1−2_Asn) (43). So, the identification of extracellular proteins that are not expected to be *O*-GlcNAc modified is a matter of concern. However, only four of the 1,271 putative *O*-GlcNAcylated proteins were considered extracellular. Nevertheless, we cannot rule out the possibility that some of the identified surface or secreted proteins might also not be *O*-GlcNAc modified.

Several identified proteins were previously described as *O*-GlcNAcylated in other models, a finding that supports the universal role of *O*-GlcNAcylation in cell biology. The 10 most abundant putative modified proteins are listed in Figure 5. As shown, tubulin is abundantly represented among protist *O*-GlcNAcylated proteins and we found it on *T. cruzi* epimastigotes by immunoprecipitation as can be seen on Figure 1C; this was expected, since *T. cruzi* microtubules are cytoskeletal structures composed of two α- and one β-tubulin isoforms and are present in the flagellum and under the plasma membrane as subpellicular microtubules, and have important functions in motility, cellular morphology, intracellular transport, and cell division. Tubulin isoforms have also been studied as a target for substances with possible antichagasic activity (44) and as vaccine antigen candidates (45). Our results suggest the possibility to influence *T. cruzi* microtubule polymerization by interfering in tubulin *O*-GlcNAcylation, given the known role of *O*-GlcNAcylation in regulating microtubule formation (46).

Notably, the detection of the 6 *O*-GlcNAc peptides (Supplementary Table 2) gave us more possible targets to study the role of the *O*-GlcNAc modification in *T. cruzi*. Trans-sialidases are a highly studied group of proteins of *T. cruzi* that are involved in pathogenesis (47). Clathrins expression has been previously reported on *T. cruzi* and are known to mediate endocytosis at the flagellar pocket and the cytostome complex (48). Helicases and DNA polymerases are linked to replication and transcription processes and even DNA repair of oxidative lesions (49). All these proteins play important roles in *T. cruzi* stress response, nutrition and life cycle progression as have been reported for mammal cells (50). The meaning of these specific *O*-GlcNAc motifs is not possible to be predicted, nevertheless, this highlights the necessity of further research to elucidate the functions of *O*-GlcNAcylation in specific proteins and cellular processes of *T. cruzi*.

## Conclusions

This is the first demonstration of the occurrence of the *O*-GlcNAc modification in *T. cruzi*. We also provide a large-scale identification of putative *O*-GlcNAcylated proteins with several cellular functions and 6 *O*-GlcNAc modification sites. Experimental evidence suggests the possible role of *O*-GlcNAcylation in the cellular homeostatic response in *T. cruzi* and opens a totally new field to study the mechanisms of parasite adaptation, survival, and invasion, which would help us to identify new drug targets.

## Data Availability

Mass spectrometry proteomics data were deposited and can be found in the ProteomeXchange Consortium via the PRIDE (25) partner repository with the dataset identifier PXD010285.

## Author Contributions

ET-G, PS-S, YP-C, and EZ developed the hypotheses. ET-G performed most of the biochemical experiments and interpreted the data. MA-G and OR-D contributed performing certain experiments. LC performed the on resin digest, the mass spectrometry analysis and provided the resulting data. MB-T, MC-B, and TL provided biological material and technical support. PS-S, YP-C, and EZ initiated the project. ET-G, YP-C, TL, EZ, and PS-S analyzed the results. ET-G, PS-S, and YP-C drafted the manuscript. All authors contributed to refining the manuscript, read and approved the final version.

### Conflict of Interest Statement

The authors declare that the research was conducted in the absence of any commercial or financial relationships that could be construed as a potential conflict of interest.

## Supplementary Material

Supplementary Figure 1 MS-MS spectra of the *O*-GlcNAcylated peptides.

Supplementary Table 1 *T. cruzi* putative *O*-GlcNAcylated proteins in blue and potential background in red. Full data are available via ProteomeXchange with identifier PXD010285.

Supplementary Table 2 *T. cruzi O*-GlcNAc peptides. Full data are available via ProteomeXchange with identifier PXD010285.
