# Supplementary material for: Identification of O-Glcnacylated Proteins in Trypanosoma cruzi
Source: Front Endocrinol (Lausanne). 2019 Mar 29;10:199. doi: 10.3389/fendo.2019.00199 (PMC6449728; doi:10.3389/fendo.2019.00199)

Peptide Summary

Sequence: AENPGLPTHFVVELPTGIRMSFK, T16-Dehydrated (-18.01057 Da)
Charge: +3, Monoisotopic m/z: 841.44397 Da (-1.73 mmu/-2.06 ppm), MH+: 2522.31736 Da, RT: 244.98 min,
Identified with: Sequest HT (v1.3); XCorr:1.99, Ions matched by search engine: 0/0
Fragment match tolerance used for search: 0.6 Da

Fragment Matches

Fragment Spectrum

Extracted from: P:\PLATEFORME_Data\180213 hupo abrf glyco\bete4ul.raw #39382 RT: 244.98
FTMS, HCD@37.50, z=+3, Mono m/z=841.44397 Da, MH+=2522.31736 Da, Match Tol.=0.9 Da

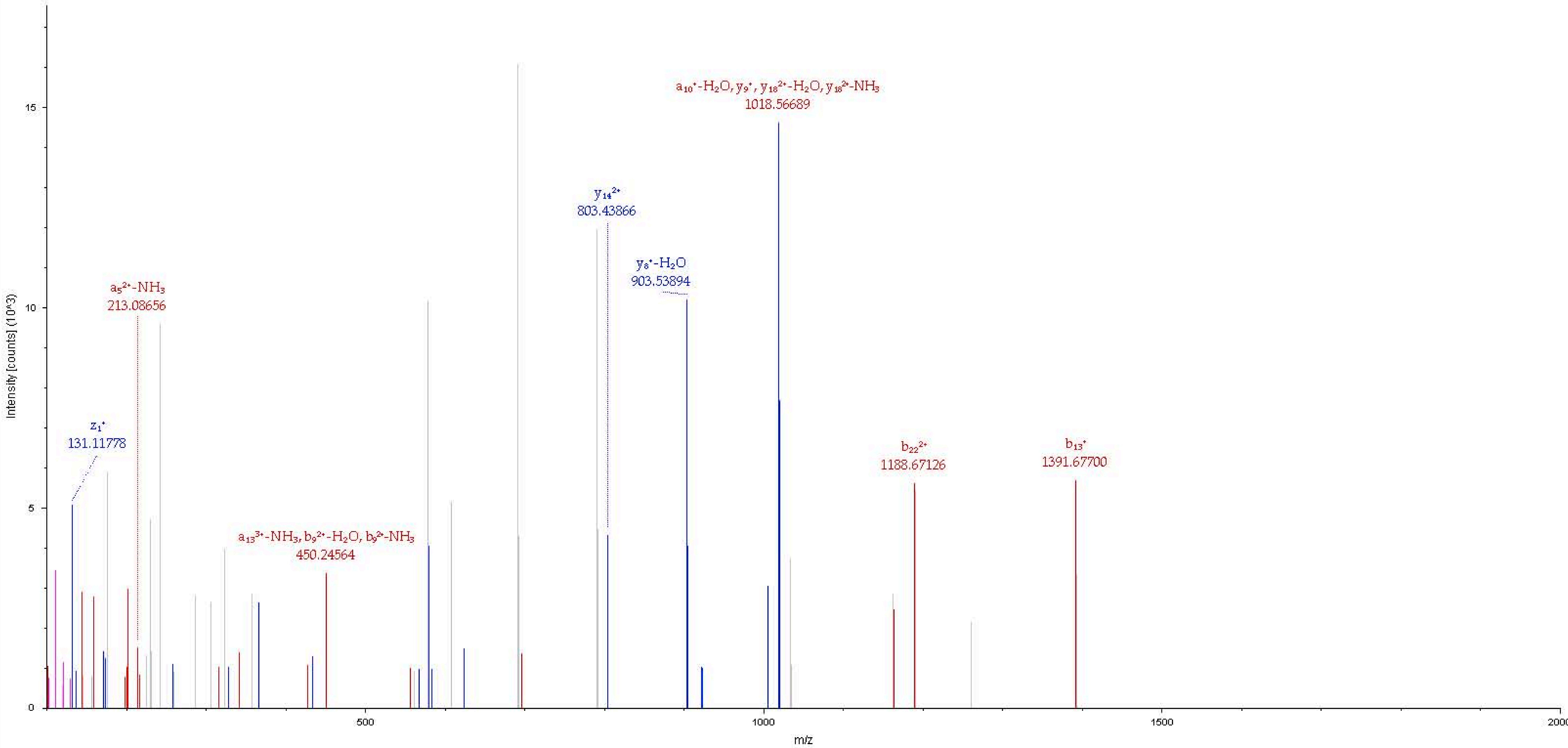

Peptide Summary

Sequence: EFFTPSSLCSPRQSR, S6-Dehydrated (-18.01057 Da), S7-Dehydrated (-18.01057 Da), C9-Carbamidomethyl (57.02146 Da)
Charge: +2, Monoisotopic m/z: 881.92462 Da (+4.47 mmu/+5.07 ppm), MH+: 1762.84197 Da, RT: 233.48 min,
Identified with: Sequest HT (v1.3); XCorr:1.79, Ions matched by search engine: 0/0
Fragment match tolerance used for search: 0.6 Da

Fragment Matches

Fragment Spectrum

Extracted from: P:\PLATEFORME_Data\180213 hupo abrf glyco\bete4ul_180214194556.raw #35865 RT: 233.48
FTMS, HCD@37.50, z=+2, Mono m/z=881.92462 Da, MH+=1762.84197 Da, Match Tol.=0.9 Da

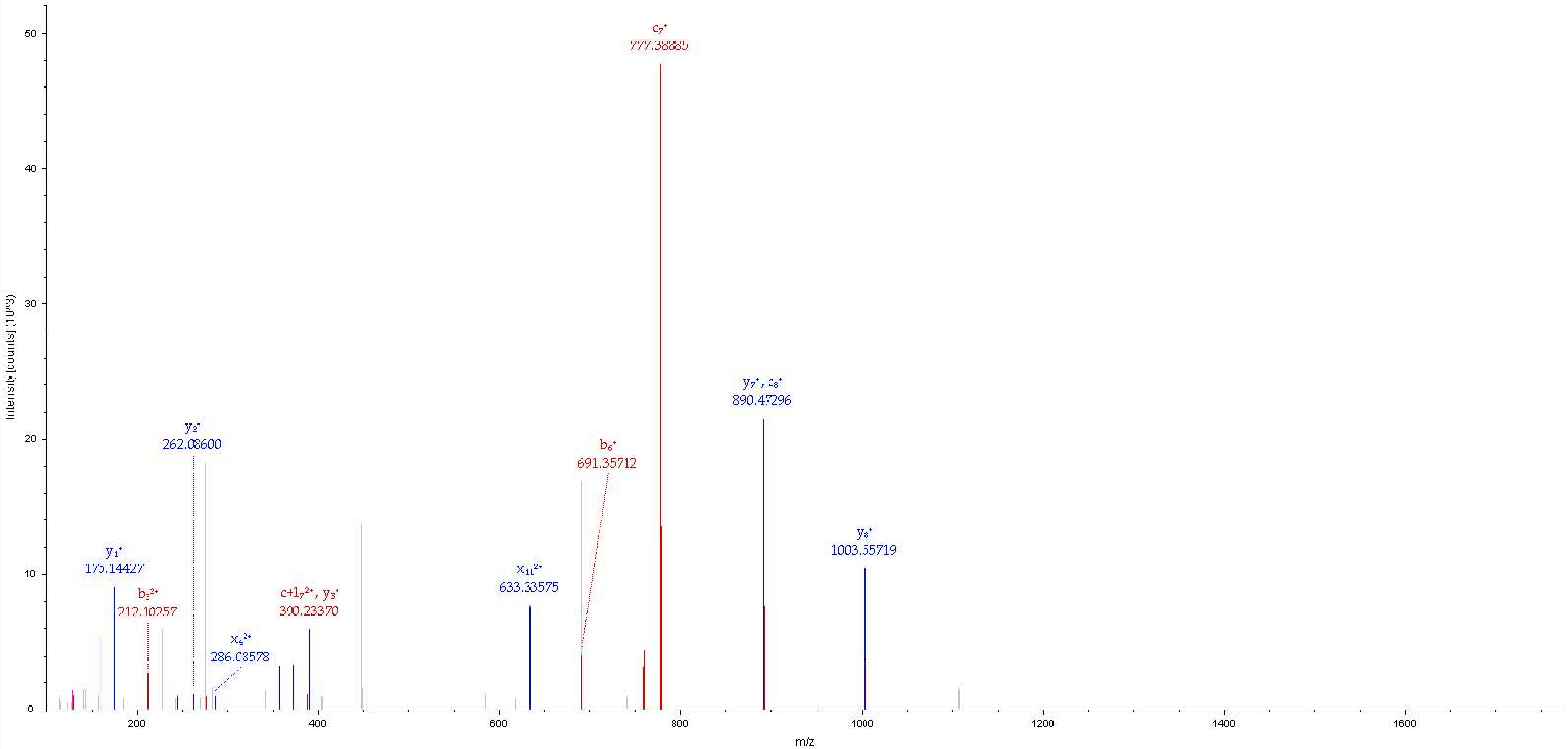

Peptide Summary

Sequence: HQVVVIGGETGSGKTTQIPQYLYEFMCESGMGGSANIVCTQPRR, C27-Cys->Dha (-33.98772 Da), M31-Oxidation (15.99492 Da), T40-Dehydrated (-18.01057 Da)
Charge: +4, Monoisotopic m/z: 1174.08118 Da (+10.78 mmu/+9.18 ppm), MH+: 4693.30288 Da, RT: 244.54 min,
Identified with: Sequest HT (v1.3); XCorr:0.98, Ions matched by search engine: 0/0
Fragment match tolerance used for search: 0.6 Da

Fragment Matches

Fragment Spectrum

Extracted from: P:\PLATEFORME_Data\180213 hupo abrf glyco\bete4ul.raw #39211 RT: 244.54
FTMS, HCD@37.50, z=+4, Mono m/z=1174.08118 Da, MH+=4693.30288 Da, Match Tol.=0.9 Da

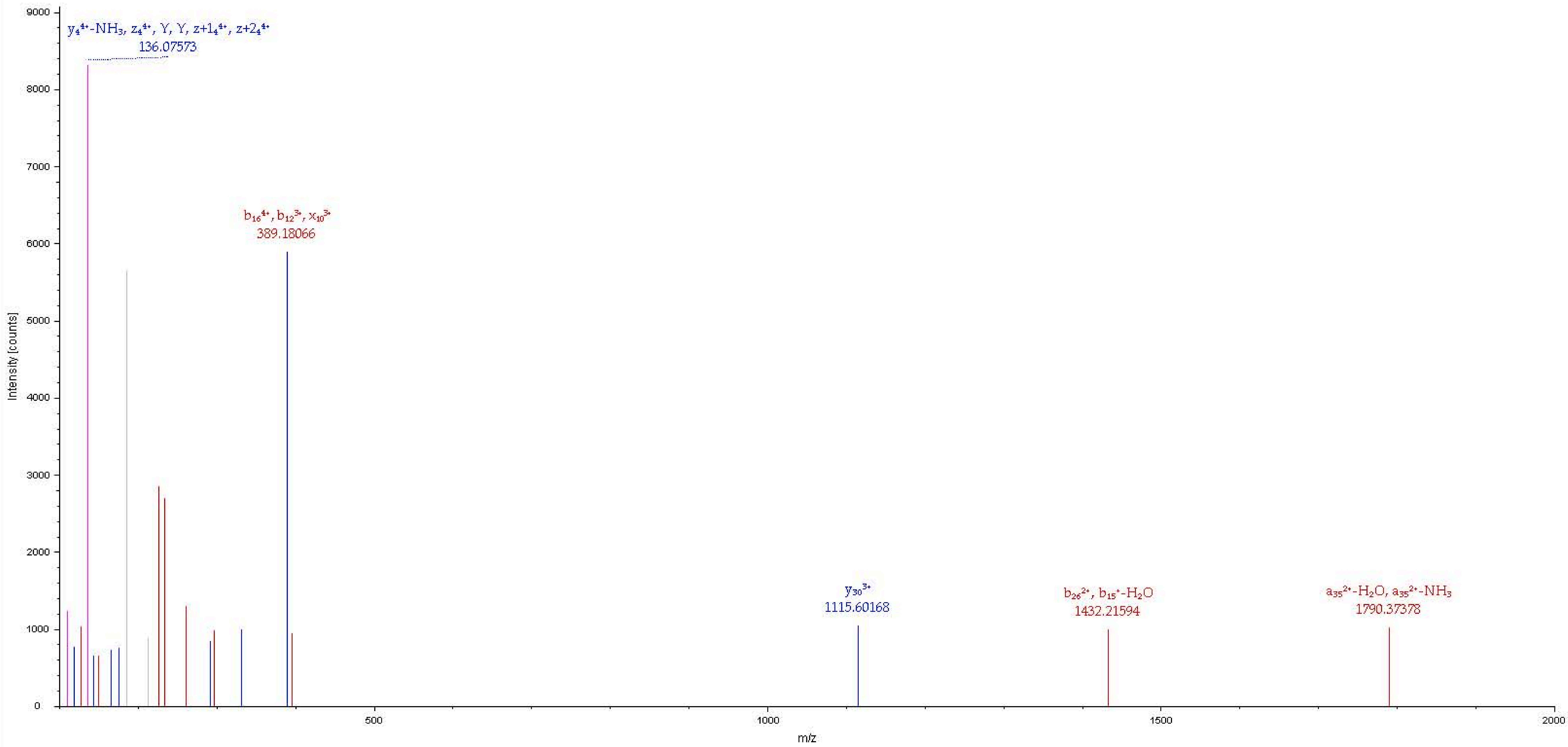

Peptide Summary

Sequence: KVDETVSMLCPSSAEK, C10-Carbamidomethyl (57.02146 Da), S13-Dehydrated (-18.01057 Da)
Charge: +2, Monoisotopic m/z: 881.92413 Da (+2.95 mmu/+3.35 ppm), MH+: 1762.84099 Da, RT: 231.73 min,
Identified with: Sequest HT (v1.3); XCorr:2.40, Ions matched by search engine: 0/0
Fragment match tolerance used for search: 0.6 Da

Fragment Matches

Fragment Spectrum

Extracted from: P:\PLATEFORME_Data\180213 hupo abrf glyco\bete4ul.raw #35831 RT: 231.73
FTMS, HCD@37.50, z=+2, Mono m/z=881.92413 Da, MH+=1762.84099 Da, Match Tol.=0.9 Da

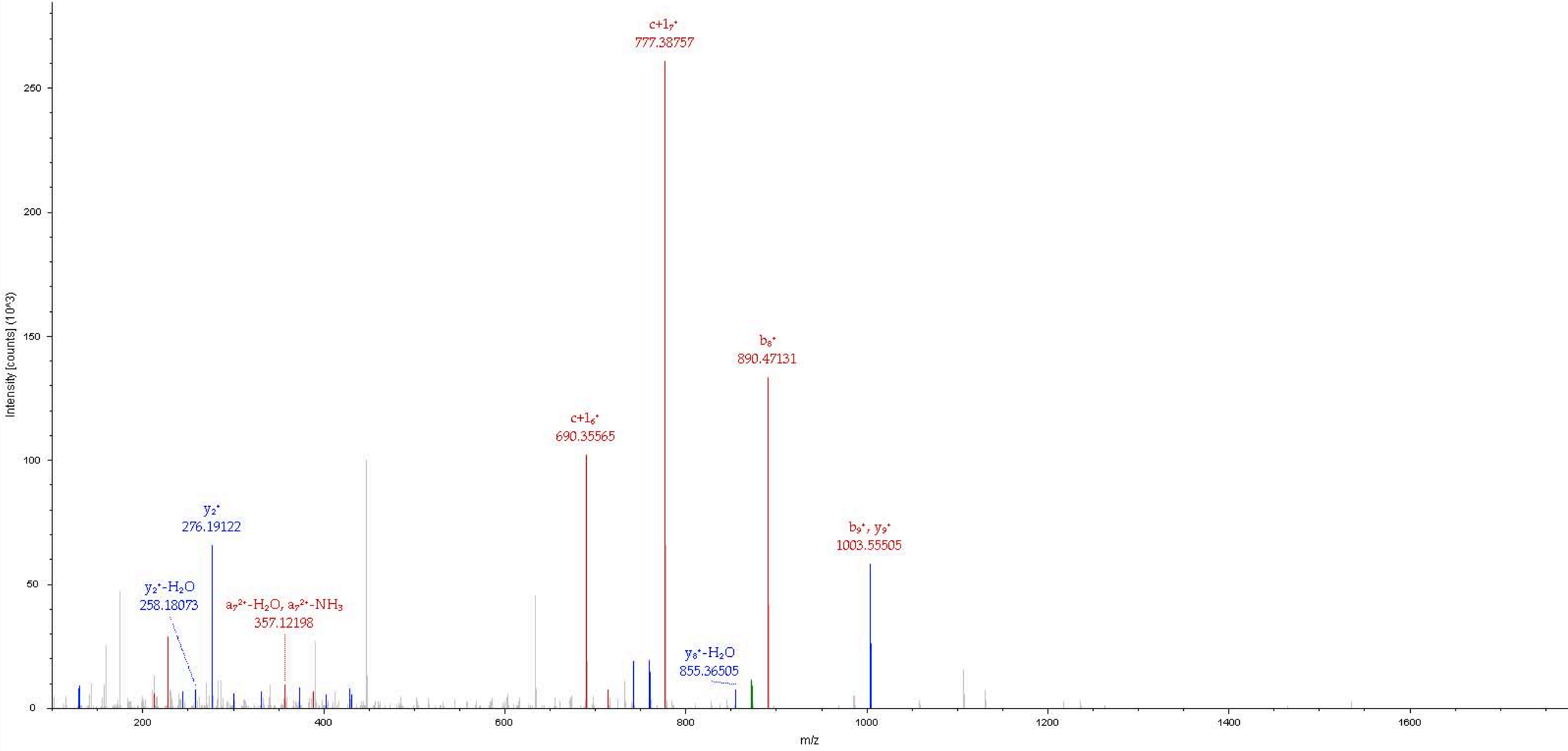

Peptide Summary

Sequence: SVAYRVSTPLR, S1-Dehydrated (-18.01057 Da)
Charge: +2, Monoisotopic m/z: 615.84552 Da (-5.81 mmu/-9.44 ppm), MH+: 1230.68376 Da, RT: 237.75 min,
Identified with: Sequest HT (v1.3); XCorr:1.17, Ions matched by search engine: 0/0
Fragment match tolerance used for search: 0.6 Da

Fragment Matches

Fragment Spectrum

Extracted from: P:\PLATEFORME_Data\180213 hupo abrf glyco\bete4ul_180214194556.raw #36895 RT: 237.75
FTMS, HCD@37.50, z=+2, Mono m/z=615.84552 Da, MH+=1230.68376 Da, Match Tol.=0.9 Da

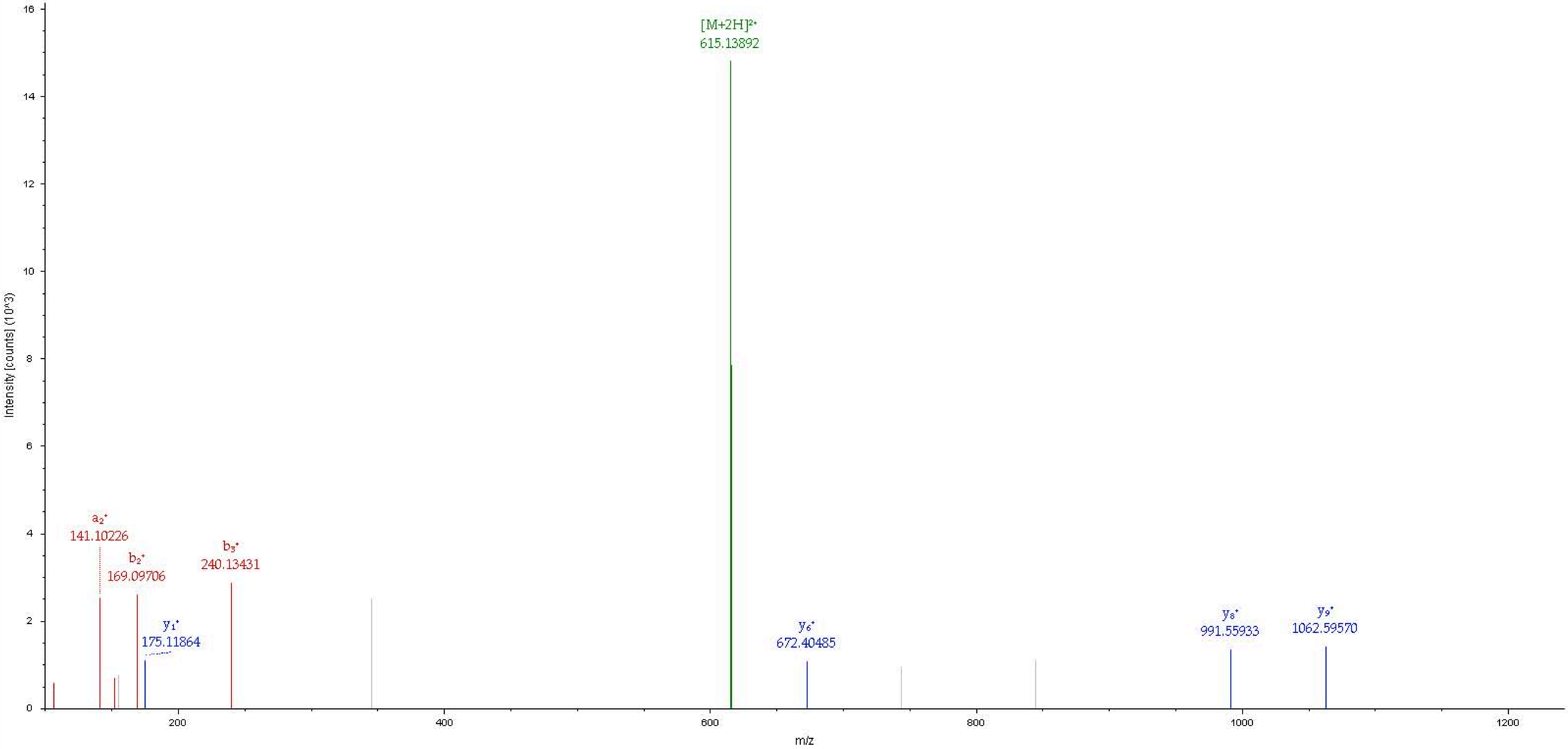

Peptide Summary

Sequence: TAASTPGAHMGVFTGMGLLLAK, T1-Dehydrated (-18.01057 Da), S4-Dehydrated (-18.01057 Da), T5-Dehydrated (-18.01057 Da), M10-Oxidation (15.99492 Da)
Charge: +2, Monoisotopic m/z: 1047.03833 Da (+1.1 mmu/+1.05 ppm), MH+: 2093.06938 Da, RT: 240.82 min,
Identified with: Sequest HT (v1.3); XCorr:0.43, Ions matched by search engine: 0/0
Fragment match tolerance used for search: 0.6 Da

Fragment Matches

Fragment Spectrum

Extracted from: P:\PLATEFORME_Data\180213 hupo abrf glyco\bete4ul.raw #38143 RT: 240.82
FTMS, HCD@37.50, z=+2, Mono m/z=1047.03833 Da, MH+=2093.06938 Da, Match Tol.=0.9 Da

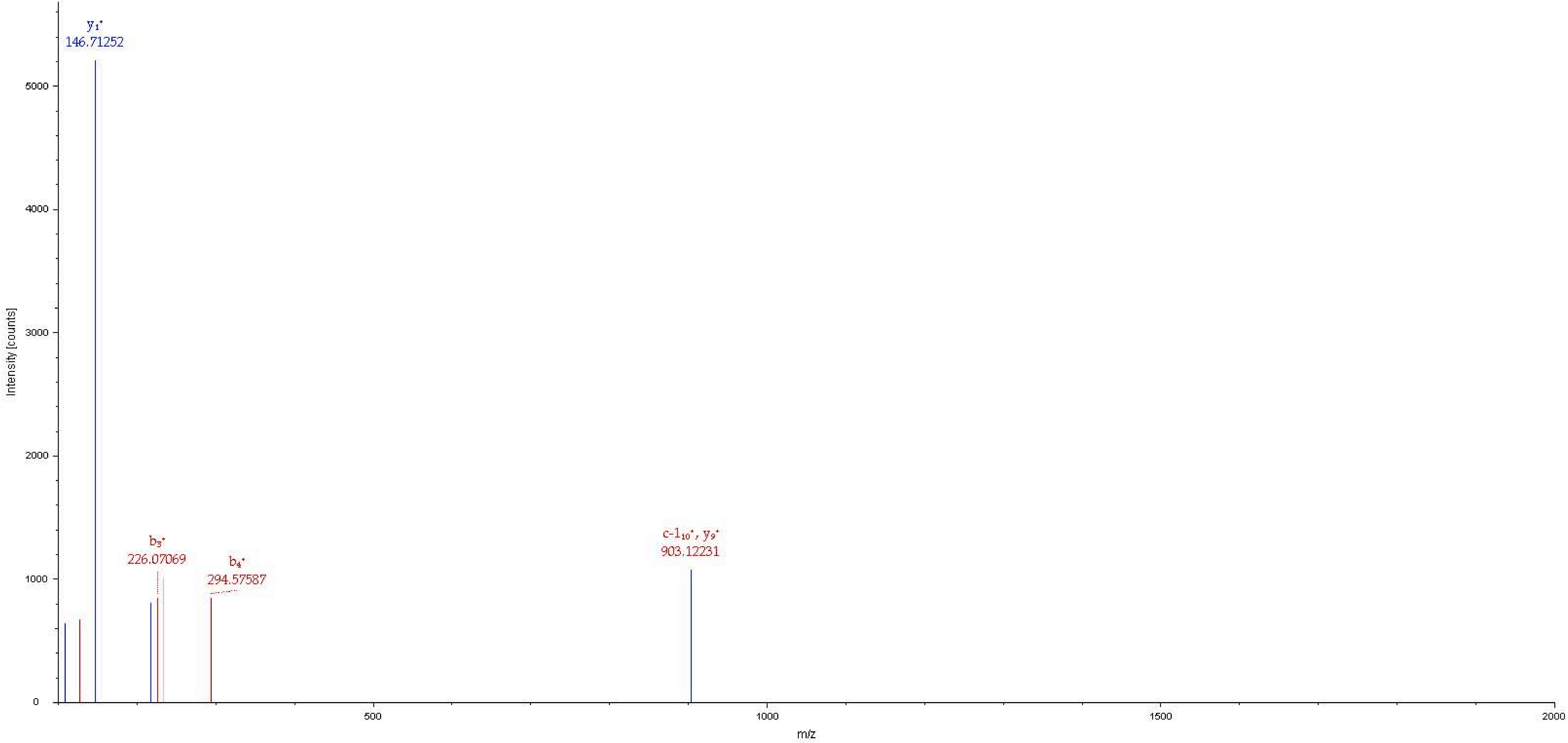

Supplement: Supplementary Figure 1 — MS-MS spectra of the O-GlcNAcylated peptides. [file Data_Sheet_1.PDF]
